# Battery Ingestion with Colonic Perforation after Colostomy Closure in a Toddler

**DOI:** 10.1055/s-0041-1741558

**Published:** 2022-03-10

**Authors:** Annamarie C. Lukish, Vivien Pat, Anisha Apte, Marc A. Levitt

**Affiliations:** 1 Division of Colorectal and Pelvic Reconstructive Surgery, Children's National Medical Center, Washington, District of Columbia, United States; 2 Division of Medicine, The George Washington University School of Medicine and Health Sciences, Washington, District of Columbia, United States; 3 Division of General Surgery, The George Washington University School of Medicine and Health Sciences, Washington, District of Columbia, United States; 4 Division of Pediatric Surgery, The George Washington University School of Medicine and Health Sciences, Washington, District of Columbia, United States

**Keywords:** disc battery ingestion, colonic foreign body, colonic perforation, COVID-19

## Abstract

Disc and button battery ingestion in children is common. In fact, data reports a dramatic increase in battery ingestion during the coronavirus disease 2019 pandemic likely as a result of increased household population density and electronic product utilization. These batteries often remain lodged in the esophagus causing potentially devastating complications if they are not removed urgently. Batteries that are passed beyond the esophagus usually do not cause any complications. We present the case of a 15-month-old male who underwent a colostomy takedown 2 months following a posterior sagittal anorectoplasty for imperforate anus. He recovered quickly, was advanced on his diet, and was discharged to home on postoperative day 3. On postoperative day 5 following the stoma closure, he presented with an acute abdomen, pneumoperitoneum and an abdominal X-ray that revealed a 21 mm disc battery in the left lower quadrant. He underwent exploration and the battery was found perforating the anastomosis. There was significant fibropurulent exudate and inflammation. The battery was removed, the anastomosis was excised, and a colostomy with Hartman's pouch was performed. The toddler recovered uneventfully.

This case offers an opportunity to discuss the concerns of battery ingestion and postoperative care following intestinal surgery in children. We could find no other similar reports in the world's literature of a disrupted colonic anastomosis due to battery ingestion.

## Introduction

Disc and button batteries are found in many consumer electronics and toys. 1 Perhaps relevant to this case, it is clear that as a result of the coronavirus disease 2019 (COVID-19) pandemic, parents, and children are sharing the home environment that is now functioning as office, classroom, daycare, and playground. 2 This scenario has likely increased the use of electronic products that may be powered by batteries. The new environment increases the risk that these batteries are left unattended and ingested by toddlers sharing the space. In the *Journal of Pediatric Gastroenterology and Nutrition* , a report documented a ninefold increase in battery ingestion during Italy's 2-month pandemic lockdown compared with the same 2-month period in the each of the 4 years prior. 3 It is reported that 50% of battery ingestions are unwitnessed, 4 and it is well known that batteries can cause significant serious injury when aspirated or swallowed. 5 Most ingestions occur in small children less than 4 years of age. Fortunately, most cases of battery ingestion (97%) have mild or no effects. 5 6 We present a child who suffered a colon perforation and anastomotic disruption of a colocolostomy by an ingested lithium disc battery that occurred at home in the postoperative period. This case represents the first report of such a complication.

## Case Report

**Fig. 1:**
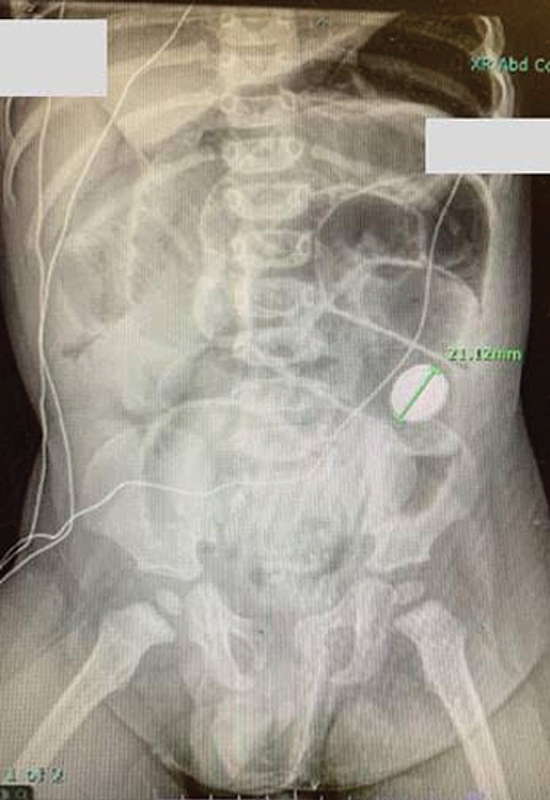
KUB X-ray on postoperative day 5. A 21 mm disc battery is located in the left lower quadrant and pneumoperitoneum is evident.

**Fig. 2:**
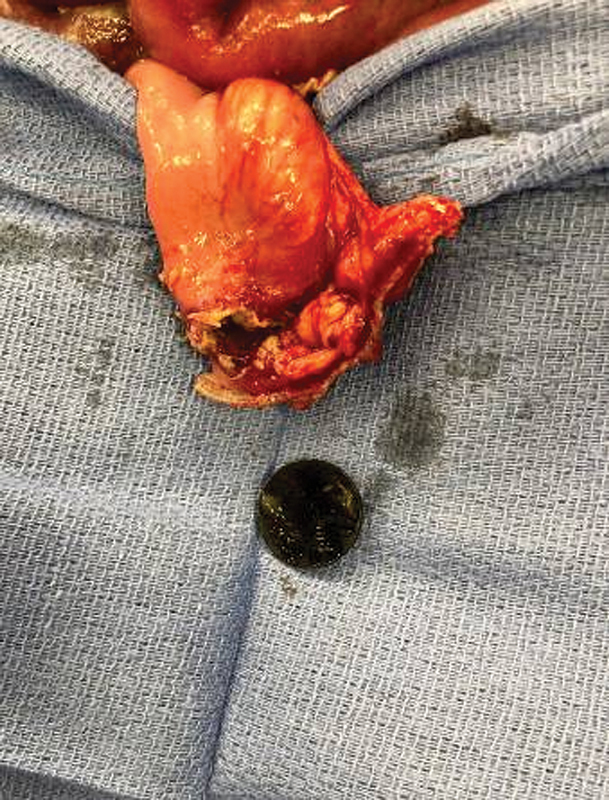
Intraoperative photo of the 21 mm disc battery where it had eroded and perforated the anastomosis. Note the significant acute and chronic inflammation of the bowel.

A 15-month-old male born with imperforate anus underwent a colostomy takedown following a posterior sagittal anorectoplasty 2 months prior. He recovered uneventfully and was discharged on a regular diet on postoperative day 3. On postoperative day 5, the patient had a fever and intolerance of feeds, followed by emesis. The mother was quick to contact our team because of the recent surgery, and she came to the emergency room within 24 hours of these symptoms. On exam, the child was in distress with abdominal distention and diffuse tenderness. His laboratory findings were consistent with a systemic inflammatory response including an elevated white blood cell count of 15.8 with a significant left shift. The differential diagnosis included postoperative abscess, anastomotic dehiscence, viral or bacterial gastroenteritis, and postoperative ileus. Acute abdominal series revealed pneumoperitoneum and a 21 mm disc battery in the left lower quadrant ( Fig. 1 ). He was resuscitated and taken to the operating room for abdominal exploration. He was found to have purulent abdominal fluid. The anastomosis was examined, and the battery was found to have perforated the anterior aspect of the colocolostomy ( Fig. 2 ). There was significant fibropurulent exudate and acute inflammation at the site. The battery was removed, and the anastomosis was excised and a colostomy with Hartman's pouch was performed. The anastomosis on pathologic evaluation had evidence of significant acute and chronic mucosal inflammation. The toddler recovered uneventfully and was discharged on postoperative day 3, and another attempt at colostomy closure is planned in 2 to 3 months.

## Discussion

A total of 55,926 disc button battery ingestions were reported to the National Poison Data System from 1985 to 2017 in children younger than 6 years of age. 7 American Poison Control Centers report over 3,300 ingestions each year. These batteries can be found in common household electronics like remote controls, hearing aids, key fobs, and cameras. When swallowed, they produce a chemical reaction that can cause serious tissue damage and can be fatal, primarily due to esophageal perforation and mediastinitis or fistulization. 4

There has been a nearly sevenfold increase in the incidence of severe morbidity and fatalities over the last decade, believed to be related to more powerful batteries needed for contemporary electronics. 8 It is possible that the increase in children and parents sharing the home environment as a result of the COVID-19 pandemic may further exacerbate these risks and concerns. 2 A recent report stating that because children are at home during the COVID-19 pandemic, certain orthopedic fractures resulting from high speed falls, trampoline, and bicycle injuries are now becoming more common having increased 12.5% compared with March, April, and May of 2019. 9 Most relevant to this case, a report by Pizzol et al stated that battery ingestion has increased in children as a result of COVID-19 lockdown in Italy. 3 Because our case has never been reported, we speculate that the pandemic may have increased the risk for this child.

Disc button batteries that traverse the esophagus often pass through the gastrointestinal tract successfully. 10 11 However, in searching the world's literature, we did find two reports of perforation of a Meckel's diverticulum as a result of such a battery impacted in the Meckel's. 12 13 We could not find any reports of colonic perforation from a battery ingestion or associated with a colo-colonic anastomosis. Logic would predict that any anatomic area of stagnation, stasis, or dysmotility would increase the risk for battery lodgment and subsequent intestinal perforation. Once the battery is lodged in tissue, injury can occur within 2 hours. 8 The injury appears to be proportional to the size and type of battery ingested, with more damage caused by the lithium batteries of the type that our patient ingested. 8 In general, children with anorectal malformations have slow colonic motility. 14 This factor coupled with the edema and narrowing associated with normal anastomotic healing likely led to battery impaction and perforation.

Prevention of ingestion in the first place is the key to avoiding such accidents in children. Health visitors and the primary health team can play a significant role in advising parents and caregivers on how to make the home a safe environment for their child. 15 It is clear that there is often anxiety for families following discharge and during the postoperative recovery of their child at home. 16 This has been compared with a type of posttraumatic stress disorder, resulting in fatigue and potential loss of focus. 17 With children at home, and with stress running high for families, it is easy for parents to let their guard down when it comes to home safety. It is plausible that during this time the child may be at increased risk of accidental ingestion.

## Conclusions

Disc and button battery ingestion is very common in children and can lead to devastating consequences. Our case of colonic perforation in the postoperative period in a toddler has never been reported and is concerning. During the COVID-19 pandemic when household population density and electronic product utilization are at a maximum, it may be prudent to provide additional guidance at discharge. Discussions with the family regarding battery and foreign body ingestion may be beneficial in avoiding a similar complication in the future.

## Notes

**New Insights and the Importance for the Pediatric Surgeon**
This case highlights the significance of battery ingestion injury in children following a colonic anastomosis. The pediatric surgeon needs to be aware of this potential risk and provide guidance to parents and caregivers during the post-operative period to mitigate this complication.
